# Identification of quantitative trait loci for kernel traits in a wheat cultivar Chuannong16

**DOI:** 10.1186/s12863-019-0782-4

**Published:** 2019-10-16

**Authors:** Jian Ma, Han Zhang, Shuiqin Li, Yaya Zou, Ting Li, Jiajun Liu, Puyang Ding, Yang Mu, Huaping Tang, Mei Deng, Yaxi Liu, Qiantao Jiang, Guoyue Chen, Houyang Kang, Wei Li, Zhien Pu, Yuming Wei, Youliang Zheng, Xiujin Lan

**Affiliations:** 1 Triticeae Research Institute, Sichuan Agricultural University, Chengdu, 611130 Sichuan China; 2 China State Key Laboratory of Crop Gene Exploration and Utilization in Southwest China, Sichuan Agricultural University, Chengdu, 611130 China; 3 College of Agronomy, Sichuan Agricultural University, Chengdu, 611130 Sichuan China

**Keywords:** Common wheat, Kernel traits, 55 K SNP array, QTL, KASP marker

## Abstract

**Background:**

Kernel length (KL), kernel width (KW) and thousand-kernel weight (TKW) are key agronomic traits in wheat breeding. Chuannong16 (‘CN16’) is a commercial cultivar with significantly longer kernels than the line ‘20828’. To identify and characterize potential alleles from CN16 controlling KL, the previously developed recombinant inbred line (RIL) population derived from the cross ‘20828’ × ‘CN16’ and the genetic map constructed by the Wheat55K SNP array and SSR markers were used to perform quantitative trait locus/loci (QTL) analyses for kernel traits.

**Results:**

A total of 11 putative QTL associated with kernel traits were identified and they were located on chromosomes 1A (2 QTL), 2B (2 QTL), 2D (3 QTL), 3D, 4A, 6A, and 7A, respectively. Among them, three major QTL, *QKL.sicau-2D*, *QKW.sicau-2D* and *QTKW.sicau-2D*, controlling KL, KW and TKW, respectively, were detected in three different environments. Respectively, they explained 10.88–18.85%, 17.21–21.49% and 10.01–23.20% of the phenotypic variance. Further, they were genetically mapped in the same interval on chromosome 2DS. A previously developed kompetitive allele-specific PCR (KASP) marker *KASP-AX-94721936* was integrated in the genetic map and QTL re-mapping finally located the three major QTL in a 1- cM region flanked by *AX-111096297* and *KASP-AX-94721936*. Another two co-located QTL intervals for KL and TKW were also identified. A few predicted genes involved in regulation of kernel growth and development were identified in the intervals of these identified QTL. Significant relationships between kernel traits and spikelet number per spike and anthesis date were detected and discussed.

**Conclusions:**

Three major and stably expressed QTL associated with KL, KW, and TKW were identified. A KASP marker tightly linked to these three major QTL was integrated. These findings provide information for subsequent fine mapping and cloning the three co-localized major QTL for kernel traits.

## Background

It is estimated that at least 2.4% of yield growth rate per year is required to meet food demand by 2050 due to the increasing world population [1]. However, crop yield increase rates have been so far unsatisfactory [2]. A better understanding and use of genetic determinants of kernel dimensions and weight could contribute to yield improvement in cereals [3]. Kernel weight is a major yield component principally defined by kernel length (KL), width (KW) and thickness [4]. Thus, it is valuable to identify and introduce favorable genes or alleles controlling kernel traits to improve yield in breeding.

Genes controlling kernel traits have been identified in tractable model species, such as *Arabidopsis thaliana* and *Oryza sativa* [5–7]. For example, *qLGY3* encoding a MADS-domain transcription factor was associated with kernel size and could be modified to increase both kernel quality and yield potential in rice [8]. *OsGW5* represents a major QTL controlling kernel width and weight in rice, and that it likely acts in the ubiquitin-proteasome pathway to control cell division during seed development [9]. The QTL *qTGW3* encodes the GSK3/SHAGGY-like kinase OsGSK5/OsSK41 and interacts with *OsARF4* to negatively regulate kernel size and weight in rice [7]. *GS9* regulates kernel shape by altering cell division and improves the appearance quality of rice [10]. Using homology cloning, several orthologous genes associated with kernel traits have been isolated and characterized in common wheat (*Triticum aestivum*, AABBDD). For instance, *TaGW2* [11] and *TaGS5* [12] were isolated in wheat based on their orthologs with *OsGW2* and *OsGS5* in rice. *TaGW2* was involved in regulation of KW, kernel weight, and kernel number in wheat [13]. *TaGS5* was associated with thousand-kernel weight (TKW) [12], and *TaGW8* was related to kernel size [14] in wheat.

In addition to isolating and characterizing wheat orthologs, numerous studies have focused on identifying quantitative trait locus/loci (QTL) for kernel traits. The detected QTL covered almost 21 chromosomes in wheat [15–20]. However, very few of them have been environmental-stably characterized and validated as the effects of QTL in hexaploid wheat are usually subtle in comparison with those identified in rice [21]. Large genetic distances between QTL and their flanking markers further restrict the utilization efficiency of kernel traits in wheat breeding.

In this study, we identified stably expressed QTL associated with KL, KW and TKW in a recombinant inbred line (RIL) mapping population developed from the cross between ‘20828’ and Chuannong 16 (‘CN16’, 2CN) based on the constructed genetic map using the Wheat55K SNP array. A kompetitive allele-specific PCR (KASP) marker tightly linked to the major and stable QTL was developed and integrated in the genetic map and could be used in molecular breeding. The results reported here laid a foundation for subsequent fine mapping and cloning the three co-localized major QTL for kernel traits.

## Results

### Phenotypic evaluation and correlation analysis

**Fig. 1:**
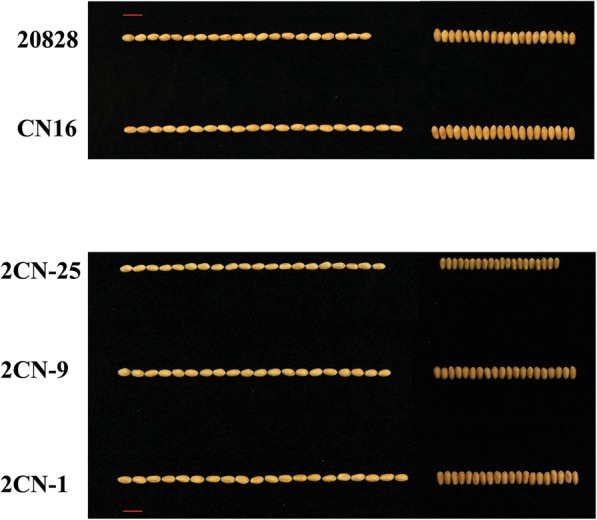
Kernel phenotypes of the parent ‘20828’, ‘CN16’ and partial RILs. The red line represents the scale = 1 cm

**Fig. 2:**
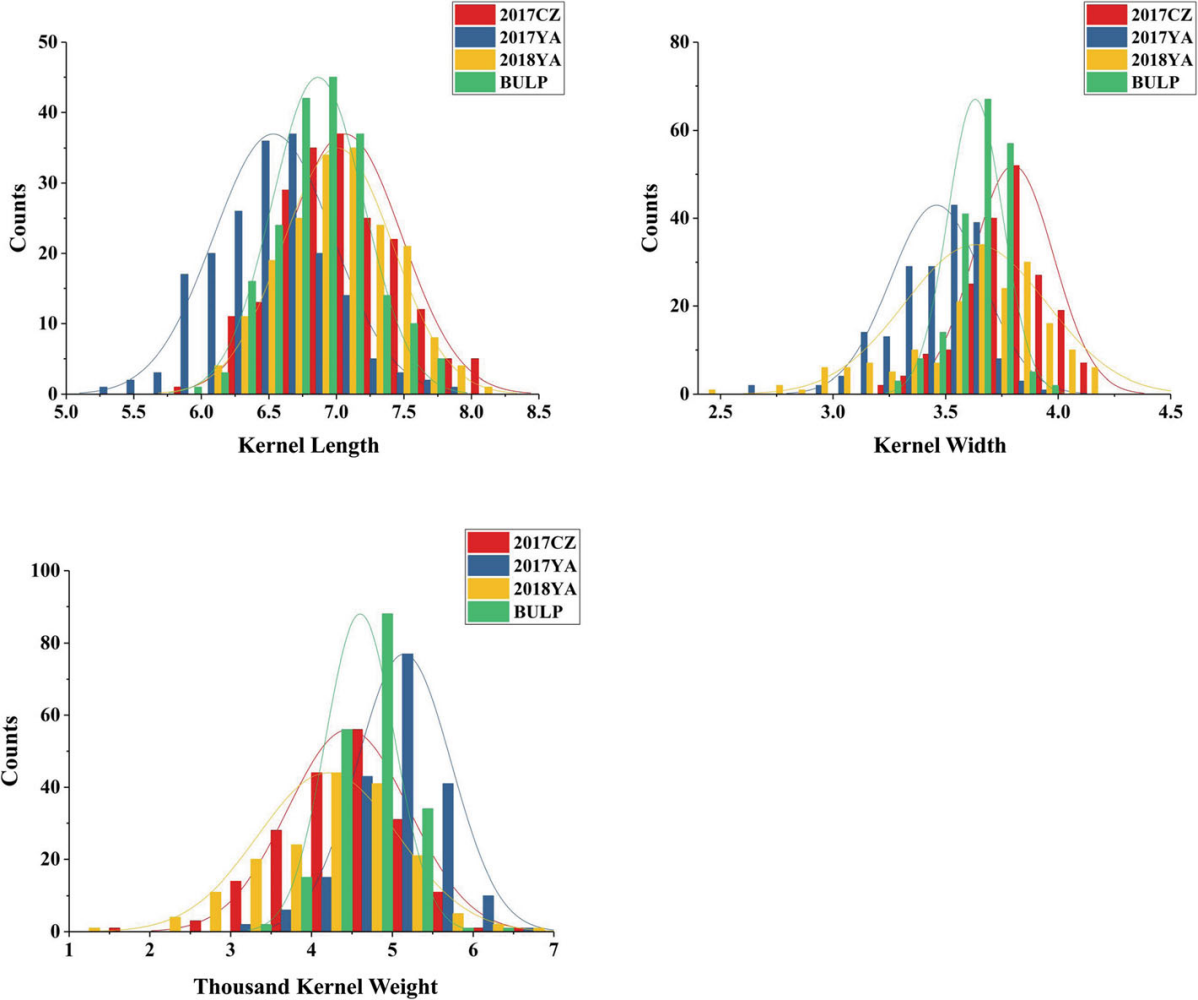
Frequency distribution of three kernel traits in 2CN population in different environments

The kernel traits of the 2CN population and their parents in different environments are listed in Table 1. ‘CN16’ had consistently and significantly higher values for KL than ‘20828’, while ‘20828’ is wider than ‘CN16’ in KW (Table 1, Fig. 1). For the 2CN population, the frequency distribution for kernel traits in all environments and best linear unbiased predictors (BLUP) showed continuous distributions with ranges from 5.33 to 8.07 mm in KL, 2.43 to 4.19 mm in KW and 10.7 to 69.3 g in TKW (Table 1, Fig. 2). The broad-sense heritability of KL, KW and TKW were 0.86, 0.64 and 0.73, respectively (Table 1).

**Table 1:** Phenotype of the parents and RILs in this study

		Parents		RIL					
Trait	Environment	20,828	CN16	Min	Max	Mean	SD	CV	*h* ^*2*^
KL (mm)	2017CZ	6.64^b^	7.16	5.97	8.07	7.06	0.42	0.06	0.86
	2017YA	6.45^b^	6.84	5.33	7.81	6.53	0.44	0.07	
	2018YA	7.17^b^	7.64	6.08	8.01	7.01	0.41	0.06	
	BLUP	6.76	7.17	5.95	7.70	6.86	0.34	0.05	
KW (mm)	2017CZ	3.84^b^	3.67	3.24	4.17	3.80	0.18	0.05	0.64
	2017YA	3.54^b^	3.51	2.64	3.94	3.46	0.20	0.06	
	2018YA	4.01^a^	3.85	2.43	4.19	3.63	0.32	0.09	
	BLUP	3.74	3.66	3.29	3.92	3.63	0.12	0.03	
TKW (g)	2017CZ	43.5	44.7	16	69.3	44.5	0.75	0.17	0.73
	2017YA	53.7^b^	51.7	31	67	51.5	0.58	0.11	
	2018YA	43.2	44.2	10.7	66.8	41.9	0.67	0.16	
	BLUP	46.6	46.6	33.7	60.8	46	0.43	0.09	

^a^ Difference is significant at the 0.05 level, ^b^ Difference is significant at the 0.01 level

Correlation analysis showed that KL, KW and TKW among different environments were all significant, and the correlation coefficients ranged from 0.38 to 0.92 (*P* < 0.01, Additional file 2: Table S1). Significant correlations with coefficients ranging from 0.48 to 0.83 among all three kernel traits based on the BLUP data were detected as well (*P* < 0.01, Additional file 3: Table S2).

Moreover, the phenotypic correlation analyses between the investigated kernel traits and other agronomic traits showed that all three kernel traits had significantly and negatively correlations with spikelet number per spike and anthesis date (*P* < 0.01). KW was significantly and positively correlated with plant height (*P* < 0.01) and significant correlations were also detected between TKW and plant height (*P* < 0.05, Additional file 4: Table S3).

### QTL mapping

**Fig. 3:**
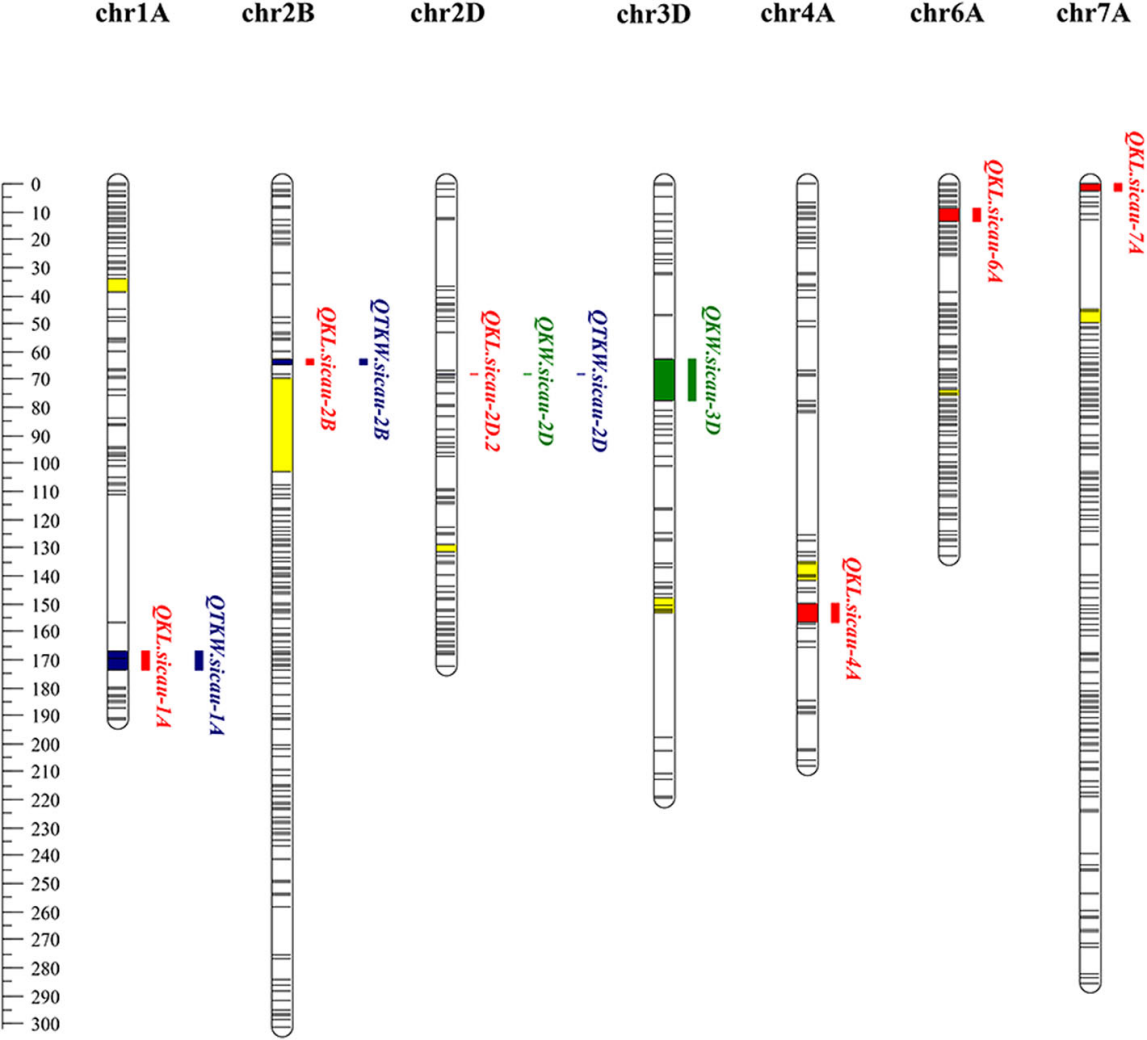
Eleven putative and stable QTL for kernel traits in the genetic map. Red color represents QTL conferring KL, green color represents QTL conferring KW, blue color represents QTL conferring TKW, and the centromere was indicated in yellow color

A total of 11 putative QTL associated with kernel traits were detected in the 2CN population (Table 2). They were located on chromosomes 1A (2 QTL), 2B (2 QTL), 2D (3 QTL), 3D, 4A, 6A, and 7A (Table 2, Fig. 3). Of them, six QTL conferring KL were identified individually explaining 2.57–18.05% of the phenotypic variance. *QKL.sicau-2D*, as a major locus, was detected in all environments and explained 10.88–18.05% of the phenotypic variance. The positive allele at *QKL.sicau-2D* was derived from ‘CN16’ (Table 2). *QKL.sicau-6A* was detected in two environments. This locus explained 5.16–7.09% of phenotypic variance. The positive allele at this QTL was derived from ‘20828’.

**Table 2:** Quantitative trait loci for kernel traits identified in the ‘20828’ × ‘CN16’ population evaluated in different environments

Trait	QTL	Environment	Chromosome	Interval (cM)	Left Marker	Right Marker	LOD	PVE (%)	Add
KL	*QKL.sicau-1A*	2017CZ	1A	167.5–172.5	*AX-111476496*	*AX-110466836*	7.12	5.21	−0.10
		2017YA		167.5–171.5	*AX-109558656*	*AX-111476496*	4.98	6.59	−0.11
		BLUP		167.5–171.5	*AX-109558656*	*AX-111476496*	5.28	4.00	−0.08
	*QKL.sicau-2B*	2018YA	2B	63.5–64.5	*AX-110425276*	*AX-111616168*	6.11	7.99	0.13
		BLUP		63.5–64.5	*AX-110425276*	*AX-111616168*	6.98	5.24	0.09
	*QKL.sicau-2D*	2017CZ	2D	67.5–68.5	*AX-111096297*	*KASP-AX-94721936*	21.13	18.05	−0.20
		2017YA		67.5–68.5	*AX-111096297*	*KASP-AX-94721936*	11.91	17.11	−0.18
		2018YA		67.5–68.5	*AX-111096297*	*KASP-AX-94721936*	8.17	10.88	−0.15
		BLUP		67.5–68.5	*AX-111096297*	*KASP-AX-94721936*	17.62	14.95	−0.15
	*QKL.sicau-4A*	2018YA	4A	157.5–158.5	*AX-110030140*	*AX-110132746*	7.70	10.00	−0.15
		BLUP		157.5–158.5	*AX-110030140*	*AX-110132746*	7.01	5.27	−0.09
	*QKL.sicau-6A*	2017CZ	6A	6.5–7.5	*AX-109498898*	*AX-111594929*	6.91	5.16	0.10
		2017YA		7.5–11.5	*AX-109498898*	*AX-111594929*	5.24	7.09	0.11
		BLUP		7.5–10.5	*AX-109498898*	*AX-111594929*	3.50	2.57	0.06
	*QKL.sicau-7A*	2017YA	7A	0–2.5	*AX-109868790*	*AX-110460191*	5.92	7.81	−0.12
		BLUP		0–1.5	*AX-109868790*	*AX-110460191*	7.26	5.40	−0.09
KW	*QKW.sicau-2D*	2017CZ	2D	67.5–68.5	*AX-111096297*	*KASP-AX-94721936*	10.35	17.21	−0.07
		2018YA		63.5–67.5	*AX-111096297*	*KASP-AX-94721936*	10.24	17.35	−0.14
		BLUP		67.5–68.5	*AX-111096297*	*KASP-AX-94721936*	14.46	21.49	−0.06
	*QKW.sicau-3D*	2018YA	3D	56.5–70.5	*AX-111126228*	*AX-110987465*	3.29	5.15	0.08
		BLUP		56.5–71.5	*AX-110987465*	*AX-110543919*	5.72	8.49	0.03
TKW	*QTKW.sicau-1A*	2017CZ	1A	167.5–171.5	*AX-109558656*	*AX-111476496*	4.59	7.13	−0.20
		BLUP		167.5–171.5	*AX-111476496*	*AX-110466836*	3.24	4.01	−0.09
	*QTKW.sicau-2B*	2017CZ	2B	63.5–64.5	*AX-110425276*	*AX-111616168*	5.30	8.25	0.22
		BLUP		63.5–64.5	*AX-110425276*	*AX-111616168*	3.81	4.80	0.10
	*QTKW.sicau-2D*	2017CZ	2D	67.5–68.5	*AX-111096297*	*KASP-AX-94721936*	7.38	11.57	−0.25
		2017YA		67.5–68.5	*AX-111096297*	*KASP-AX-94721936*	9.57	13.30	−0.23
		2018YA		67.5–68.5	*AX-111096297*	*KASP-AX-94721936*	6.77	10.01	−0.32
		BLUP		67.5–68.5	*AX-111096297*	*KASP-AX-94721936*	16.38	23.2	−0.21

Two QTL conferring KW, *QKW.sicau-2*D and *QKW.sicau-3D* were detected and accounted for 5.15–21.49% of the phenotypic variance. *QKW.sicau-2D* was a major locus and explained 17.21–21.49% of the phenotypic variance in three environments and the BLUP dataset. The positive allele at it was derived from ‘CN16’ (Table 2).

Three QTL associated with TKW were detected with 4.01–23.20% of the phenotypic variance explained. A major QTL, *QTKW.sicau-2D*, accounting for 10.01–23.20% of the phenotypic variance, was stably detected in three environments and the BLUP dataset. The positive allele at this QTL was contributed by ‘CN16’ (Table 2).

The remaining 8 QTL including *QKL.sicau-1A*, *QKL.sicau-2B*, *QKL.sicau-4A*, *QKL.sicau-6A*, *QKL.sicau-7A*, *QTKW.sicau-1A*, *QTKW.sicau-2B* and *QKW.sicau-3D* explained less than 10% of the phenotypic variance and could only be detected in less than two environments (Table 2).

Interestingly, the three major and stable QTL, *QKL.sicau-2D* for KL, *QKW.sicau-2D* for KW, and *QTKW.sicau-2D* for TKW, were co-located in the same interval between 67.5 and 68.5 cM. *QKL.sicau-1A* for KL and *QTKW.sicau-1A* for TKW were co-located in the interval between 167.5 and 172.5 cM. *QKL.sicau-2B* for KL and *QTKW.sicau-2B* for TKW were also co-located in the interval between 63.5 and 64.5 cM (Table 2, Fig. 3). These three co-located QTL intervals suggest that there may be a major QTL with pleiotropic effects affecting related traits or a cluster of linked QTL that affect multiple various traits [22, 23].

QTL × environment (QE) interaction analysis showed that a total of 45 QTL were detected (Additional file 5: Table S4). Eleven of these QTL were the same as those detected in individual environment QTL mapping. For instance, *QKL.sicau-2D*, *QKW.sicau-2D* and *QTKW.sicau-2D* were all detected, further indicating that they were major and stable. The remaining QTL showed low LOD scores and low phenotypic variance explained in the QE interaction analysis.

### Marker development and validation

**Fig. 4:**
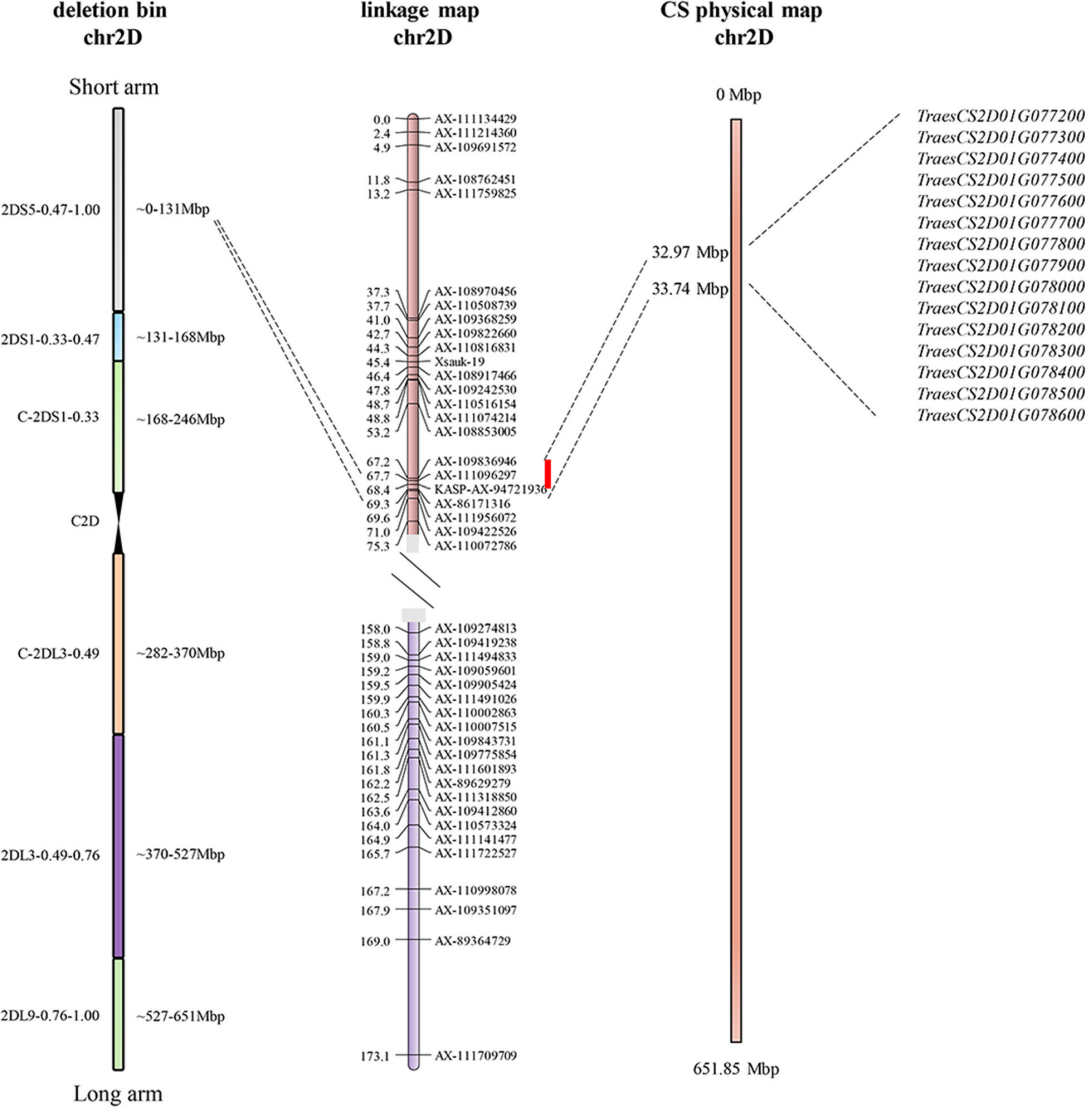
Genetic/physical maps and the predicted genes in the target interval for the three major QTL on ‘CS’

The co-located interval for the three major QTL, *QKL.sicau-2D*, *QKW.sicau-2D* and *QTKW.sicau-2D* were firstly mapped between *AX-111096297* and *AX-86171316* (Fig. 4). SNPs in this region detected by the Wheat660K array in the parents of the 2CN population were converted to KASP markers [24]. *KASP-AX-94721936* was genetically mapped between *AX-111096297* and *AX-86171316* (Fig. 4). Finally, QTL re-mapping showed that *QKL.sicau-2D*, *QKW.sicau-2D* and *QTKW.sicau-2D* were all located between *AX-111096297* and *KASP-AX-94721936* (Fig. 4). In addition, *AX-111096297* and *KASP-AX-94721936* were used to BLAST against the pseudomolecules of ‘Chinese Spring’ (‘CS’). BLAST results showed that *AX-111096297* and *KASP-AX-94721936* were located at 32.97Mbp and 33.74Mbp, respectively, in the deletion bin of 2DS5–0.47-1.00 on the short arm of chromosome 2DS (Fig. 4).

**Fig. 5:**
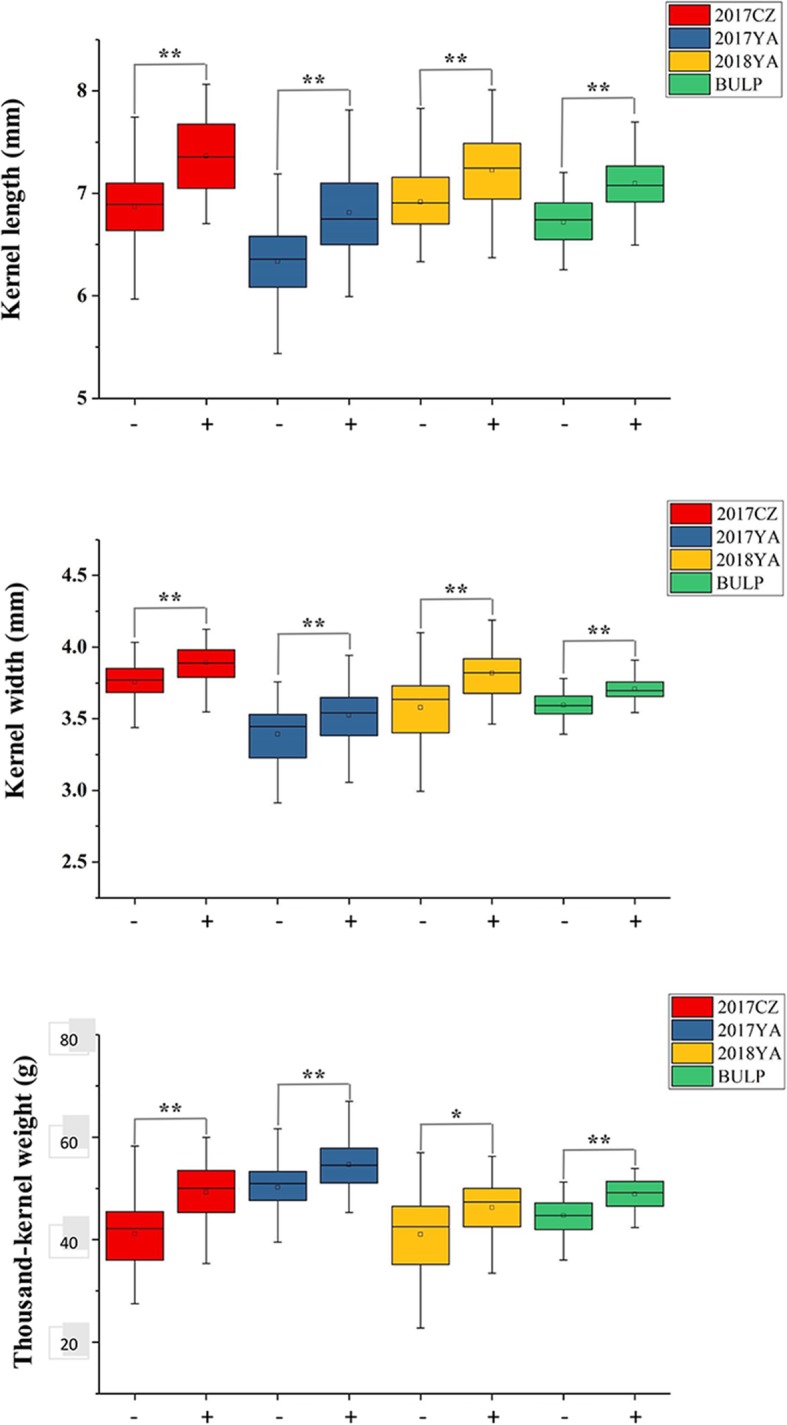
Effects of *QKL.sicau-2D*, *QKW.sicau-2D* and *QTKW.sicau-2D* in 2CN population. ‘-’ represents the homozygous lines of ‘20828’ allele, ‘+’ represents the homozygous lines of ‘CN16’ allele, * Significance at the 0.05 probability level, **Significance at the 0.01 probability level

The homozygous lines of parental alleles ‘20828’ and ‘CN16’ at each *QKL.sicau-2D*, *QKW.sicau-2D* and *QTKW.sicau-2D* were selected based on the genotyping data of *AX-111096297* and *KASP-AX-94721936* for the 2CN population. *T*-test showed that the lines carrying the ‘CN16’ alleles had significantly higher phenotypic values than those carrying the ‘20828’ alleles at all the three QTL in different environments and the BLUP datasets (*P* < 0.05, Fig. 5).

### Detection of the effect of 1BL/1RS translocation on kernel traits

No significant difference was detected between lines carrying 1BS and 1RS for KL, KW and TKW (Additional file 1: Figure S1). This result likely indicated that there is no QTL affecting these kernel traits on the 1BS or 1RS chromosome arm.

## Discussion

*Aegilops tauschii*, as the donor of the D subgenome, provides an important genetic resource for hexaploid wheat [25–27]. Zhao et al. [28] integrated a large amount of key agronomic genes/QTL and anchored them to the physical map of *Ae. tauschii*. Their results suggested that the D genome, especially for chromosomes 2D and 7D, has made strong positive contributions to wheat improvement. Thus, studies on the D genome are meaningful for understanding evolution and domestication of wheat. Numerous studies have revealed a large number of QTL for kernel traits on 2D [16, 19, 20, 29–36]. Here, three major stable QTL, *QKL.sicau-2D*, *QKW.sicau-2D* and *QTKW.sicau-2D*, associated with KL, KW and TKW, respectively, were detected on the short arm of chromosome 2DS. These results further indicated that chromosome 2D likely contributed positively to kernel traits and yield.

Comparison of physical intervals showed that the major QTL *QKL.sicau-2D* and *QKW.sicau-2D* were mapped in different intervals from those detected previously on chromosome 2D (Additional file 6: Table S5). However, we found that *QTKW.sicau-2D* was co-located with the locus for TKW reported by Yu et al. [19], suggesting they are likely alleles. Given the co-located of the three major QTL for KL (*QKL.sicau-2D*), KW (*QKW.sicau-2D*), and TKW (*QTKW.sicau-2D*) in this study, *QTKW.sicau-2D* may have a different role from that reported in Yu et al. [19]. These three major QTL were mapped in the same region between 32.97Mbp and 33.74Mbp covering 0.77 Mbp. There were 15 genes in this interval, and 11 of them were likely associated with kernel traits (Additional file 7: Table S6, Fig. 4). For example, *TraesCS2D01G077400* encodes an actin cross-linking protein and was detected to be mainly expressed in pollen of *Arabidopsis* [37], thus likely affecting kernel development. *TraesCS2D01G077900* encodes a DnaJ domain containing protein and reacts on the polar nuclear fusion further affecting endosperm proliferation in *Arabidopsis thaliana* [38].

In addition to the major QTL, we also identified a few minor QTL expressed in a single or two environment(s). These minor and unstable QTL could be mostly affected by environmental factors and may not be always expressed. The co-located interval (581.42 Mbp-584.83 Mbp on 1AL) for *QKL.sicau-1A* and *QTKW.sicau-1A* was overlapped with the co-located cluster for *QKl.ncl-1A.1* and *QTkw.ncl-1A.1* [39], suggesting they may be allelic (Additional file 6: Table S5). There were 73 predicted genes in this interval (Additional file 7: Table S6). The co-located interval (67.47 Mbp-72.59 Mbp on 2BS) for *QKL.sicau-2B* and *QTKW.sicau-2B* was overlapped with *QTkw-2B.3* [20] and *QTgw.crc-2B* [29] for TKW, indicating they may be alleles. There were 43 predicted genes in this interval (Additional file 7: Table S6). *QKL.sicau-7A* was overlapped with *QGl.cau-7A.1* [11] and the locus flanked by *wPt-0321* and *Xbarc121* [40], and thus they were likely alleles (Additional file 6: Table S5). There were 21 predicted genes in this interval (8.24 Mbp – 8.39 Mbp on 7AS). *QKL.sicau-4A* and *QKL.sicau-6A* were not overlapped with previously identified QTL for KL, suggesting they might be new loci (Additional file 6: Table S5). There were 131 and 71 predicted genes, respectively, in their located intervals (41.74 Mbp – 60.36 Mbp on 4AL and 6.27 Mbp-9.48 Mbp on 6AS). Few QTL for KW have been reported, and comparison of *QKW.sicau-3D* with those identified in previous studies showed no overlapped intervals. There were 80 predicted genes in this interval (573.10 Mbp - 578.25 Mbp on 3DL). For the predicted genes in the intervals of these minor and unstable QTL, a few were involved in growth and development of kernel. For instance, *TraesCS7A01G129200* encodes an F-box family protein. F-box protein is known to be involved in the nutrient and reproductive growth and development of many plants, and can function as a site of protein-protein interaction providing a basis for grain grouting [41]. *TraesCS3D01G474800* encodes an expansin protein. Previous studies showed that expansin proteins are cell wall proteins [42], and they can regulate plant growth through controlling cell extension via the disruption of hydrogen bonds between matrix glucans and cellulose. *TraesCS6A01G015200* encodes a mitochondrial transcription termination factor, which can promote embryo and endosperm development, resulting in large kernels [43].

In the present study, although the KW value of ‘CN16’ was lower than that of ‘20828’, we detected one major QTL *QKW.sicau-2D* at which the positive allele was contributed from ‘CN16’ and only a minor QTL *QKW.sicau-3D* with lower explained phenotypic variance at which the positive allele was contributed from ‘20828’. Similar findings are not uncommon. In previous QTL analysis, positive effects at QTL have frequently been contributed by the lower-value parents. For example, although the phenotypes of KW and TKW in parents of YN15 and SJZ54 were lower than those of M8008, the effects of the identified QTL for KW and TKW were increased by YN15 and SJZ54 alleles [44]. Breseghello and Sorrells [45] identified a major QTL on chromosome 2D for grain weight linked with SSR marker *wmc18*. Despite the parent AC Reed showed larger seeds than the other parent Grandin, the Grandin allele at *wmc18* was responsible for an increase of approximately 1.5 mg kernel^− 1^ [45]. The parent ‘20828’ likely possesses more than one allele that contributes to the formation of wider kernel. As the low coverage of SNP array around the centromere of chromosomes may lead to the lack of mapped markers on these genetic regions [46]. Thus, we cannot rule out the possibility that other QTL for KW at which the positive alleles are from ‘20828’ might be located around the centromere where the genetic map was absent in this study.

In this study, positive and significant correlations among all the three kernel traits were detected (Additional file 3: Table S2). Similar results were reported in previous studies [35, 39, 45]. This suggests that selection for larger kernels was accompanied by selection for heavier kernels during domestication and breeding process [39]. KL, KW and TKW were all significantly and negatively correlated with spikelet number per spike and anthesis date (Additional file 4: Table S3). QTL mapping indicated that major QTL for spikelet number per spike [24] and anthesis date were co-located with the major QTL for KL, KW and TKW (Additional file 8: Table S7), further confirming their close relationships. It is well known that for a single spike, an increase in spikelet number is usually accompanied with reduced kernel weight due to nutrition competition [47–49]. This was also clearly manifested by the reciprocal action of the parental alleles at a co-located interval for spikelet number per spike and TKW (Additional file 8: Table S7). The alleles from ‘20828’ increased spikelet number per spike, while the corresponding alleles from ‘CN16’ increased TKW. TKW and KW were significantly and positively correlated with plant height (Additional file 4: Table S3). The physical positions of *QKW.sicau-2D* and *QTKW.sicau-2D* were far away from the dwarfing gene *Rht8* [50] for plant height on the physical map of ‘CS’. Thus, there may be a potentially other pleiotropic locus controlling TKW and plant height as indicated by QTL mapping (Additional file 8: Table S7).

Functional markers have been effectively applied in some breeding programs [51, 52]. Molecular markers should possess the feature of high-throughput and cost-effectiveness [53]. With the decrease of sequencing cost, a large amount of SNPs have been identified. Given its advantages, KASP marker has been widely applied in wheat genetics and breeding. Here, the developed KASP marker will be helpful for further selection of heterozygous lines for developing near-isogenic lines and QTL validation in different backgrounds.

## Conclusion

In this paper, we identified three major and stably expressed and eight minor QTL associated with kernel traits based on the linkage map constructed by the Wheat55K SNP array. Three co-located intervals for kennel traits were identified. One was located on the short arm of chromosome 2D containing the three major and likely novel QTL conferring KL, KW and TKW, respectively. The other two both containing minor QTL for KL and TKW were located on chromosomes 1A and 2B, respectively. A few genes involved in regulation of kernel growth and development were identified in the intervals of these identified QTL. A KASP marker tightly linked the three major QTL would be useful for subsequent fine mapping and molecular marker selection breeding.

## Methods

### Plant materials and field environments

A RIL mapping population containing 199 F_6_ lines was developed from the cross between ‘20828’ and ‘CN16’. ‘CN16’ is a commercial cultivar with strong tillering and suitable plant type. The line ‘20828’, with high level of resistance to rust, has been widely utilized as a crossing parent in wheat breeding.

The 2CN population was planted at Chongzhou (103° 38′ E, 30° 32′ N) in 2017 (2017CZ) and Ya’an (103°0′E, 29°58′ N) in 2017 and 2018 (2017YA and 2018YA) in a randomized block design. Each line was single-seed planted in one row of 2 m in length with 10 cm between plants within a row and 30 cm between rows. Nitrogen and superphosphate fertilizers were applied at a rate of 80 and 100 kg/ha, respectively, at sowing [19]. Field management was performed according to the common practices for wheat production. At least 3 main spikes of different plants in each line were harvested when ripening.

### Phenotypic data

Thirty kernels of each line were scanned by Epson Expression 10,000 XL. KL and KW were evaluated by WinSEEDLE (Regent Instruments Canada Inc) based on the output images. TKW was calculated as 10 folds of the weight of 100 seeds with three replicates. The other agronomic traits, including spikelet number per spike, spike length, plant height, productive tiller number, kernel number per spike and anthesis date, were investigated with five plants of each line at the corresponding stage as described in previous studies [24, 46]. Details of investigated traits in different environments were listed in Additional file 9: Table S8. SPSS 22 (IBM SPSS, Armonk, NY, USA) was used for analyzing the phenotypic variance. SAS V8.0 (SAS Institute, Cary, North Carolina) was used for calculating the BLUP for all the investigated traits from different environments. The Pearson correlations between various investigated traits based on the BLUP dataset and between different environments were calculated using SPSS 22. The broad-sense heritability (*h*^*2*^) across different environments was estimated as described by Smith, et al. [54]. Student’s *t*-test (*P* < 0.05) performed by SPSS 22 was used to estimate the significant differences between two parents for three kernel traits.

### Map construction and QTL mapping

The previously constructed genetic map of 2CN population [46, 55] consisted of 34 linkage groups spanning 3005.04 cM and covered all 21 chromosomes of wheat. Here, we integrated the 34 linkage groups into 21 groups covering each of the 21 chromosomes of wheat. The re-constructed genetic map contained 2513 bin markers. The average interval between two adjacent markers is 1.74 cM. The A, B, and D subgenomes were 1483.88, 1513.80 and 1372.33 cM, with a density of 1.50, 1.53, and 2.58 cM/marker, respectively (Additional file 10: Table S9).

QTL mapping was performed using IciMapping 4.1 based on inclusive composite interval mapping (ICIM). The presence of a QTL was detected above a 3.0 log-of-odds (LOD) threshold. The QE interaction was calculated using data from all the three environments by IciMapping 4.1 with pre-adjusted parameters: Step = 1 cM, PIN = 0.001, and LOD = 3.0. QTL explained more than 10% of phenotypic variance and detected in more than 3 environments were considered to be major QTL. QTL were named according to the rules of International Rules of Genetic Nomenclature (http://wheat.pw.usda.gov/ggpages/wgc/98/Intro.htm). ‘KL’, ‘KW’, ‘TKW’ and ‘sicau’ represent ‘kernel length’, ‘kernel width’, ‘thousand-kernel weight’ and ‘Sichuan Agricultural University,’ respectively.

### Molecular marker analysis

For KASP marker development, the whole genomic DNA of the parents for the 2CN population was collected by using the Hi-DNAsecure Plant Kit (Tiangen Biotech Beijing co., Ltd) and further hybridized on the Wheat660K SNP (630, 517) genotyping array by CapitalBio Technology Company (Beijing) as descripted previously [24]. Based on genotyping results, a KASP marker was developed in putative QTL regions following standard KASP guidelines (https://www.lgcgroup.com/LGCGroup/media/PDFs/Products/Genotyping/KASP-genotyping-chemistry-User-guide.pdf). The allele-specific forward primers were designed carrying the standard FAM (5′GAAGGTGACCAAGTTCATGCT 3′) and HEX (5′ GAAGGTCGGAGTCAACGGATT 3′) tails with the targeted SNP at the 3′ end. A common reverse primer was designed with the total amplicon length was 71 bp. The detailed primers are listed in Additional file 11: Table S10. Moreover, *KASP-AX-94721936* was utilized for genotyping 2CN population. Ten μL PCR reaction mixtures contained 5 μl of 1× KASP master mixture, 50 ng of genomic DNA, 3.1 μl ddH_2_O and 1.4 μl primer mixture (comprised by 30 μl reverse primer, and 12 μl of each forward primer and 40 μl ddH_2_O). The PCR cycling parameters were: hot start at 94 °C for 15 min, followed by ten touchdown cycles (94 °C for 20 s; touchdown at 61 °C initially and decreasing by − 0.6 °C per cycle for 1 min), followed by 25 additional cycles of annealing (95 °C for 20 s; 55 °C for 1 min). The whole process was carried on real-time PCR (BioRad®, CFX-96) system. The difference between the homozygous lines of two parental alleles based on the genotyping results was detected using student’s *t*-test (*P* < 0.05) with SPSS 22.

### Comparison of QTL related to kernel traits

The genome assembly and coding sequences (CDS) of the wheat cultivar Chinese Spring or ‘CS’ [IWGSC RefSeq v1.0] [56] were download from https://urgi.versailles.inra.fr/download/iwgsc/. We used flanking markers of major QTL to BLAST against the pseudomolecules of ‘CS’ to get their corresponding physical positions. Genes in the target region were retrieved based on CDS (IWGSC_RefSeq_Annotations_v1.0 for ‘CS’) and were analyzed on UniProt (http://www.uniprot.org/) for annotation and function.

### Estimation of effect of 1BL/1RS translocation on kernel traits

As ‘CN16’ carries the 1BL/1RS translocation [57], the 1BL/1RS translocation of the 2CN RILs derived from ‘20828’ and ‘CN16’ were previously identified based on the genotype of SNP markers on chromosome 1BS [46]. As nearly no genetic recombination occurred between 1RS and 1BS, the constructed genetic map did not cover 1BS [55]. We thus estimated the possible effect of 1BL/1RS translocation on kernel traits of 2CN population. The previous identified lines carrying 1RS (34 lines) and 1BS (139 lines), respectively [46], were compared using student’s *t*-test (*P* < 0.05) with SPSS 22.

## Supplementary information

**Additional file 1:** **Figure S1.** Effect of 1BL/1RS translocation on kernel traits. ‘-’ represents the homozygous lines carrying 1BS, ‘+’ represents the homozygous lines carrying 1RS, N represents no significant difference were detected.

**Additional file 2:** **Table S1.** Correlation coefficients for kernel traits in different environments.

**Additional file 3:** **Table S2.** Correlation coefficients among different kernel traits.

**Additional file 4:** **Table S3.** Correlation coefficients between agronomic traits and kernel traits in 2CN population.

**Additional file 5:** **Table S4.** Quantitative trait loci detected in the QTL × environment interaction module.

**Additional file 6:** **Table S5.** Comparison of QTL for kernel traits-related genes from different chromosomes identified in this study with those in previous studies.

**Additional file 7:** **Table S6.** Predicted genes in the interval of the QTL identified in this study.

**Additional file 8:** **Table S7.** QTL mapping for plant height (PH), anthesis date (AD), and spikelet number per spike (SNS) on chromosome 2D.

**Additional file 9:** **Table S8.** Details of investigated traits in different environments.

**Additional file 10:** **Table S9.** Genetic map of the 2CN mapping population.

**Additional file 11:** **Table S10.** Details of KASP markers.

## Data Availability

The datasets supporting the conclusions of this study are included in this published article and its supplementary information files.

## Abbreviations

‘CN16’: Chuannong16
‘CS’: ‘Chinese Spring’
AS-PCR: Allele-specific PCR
BLUP: Best linear unbiased predictors
CAPS: Cleaved amplified polymorphic sequences
CDS: Coding sequences
CZ: Chongzhou
dCAPS: Derived cleaved amplified polymorphic sequences
KASP: Kompetitive allele-specific PCR
KL: Kernel length
KW: Kernel width
QTL: Quantitative trait locus/loci
RIL: Recombinant inbred line
SNP: Single nucleotide polymorphism
TKW: Thousand-kernel weight
YA: Ya’an
