# Supplementary figures and images for: Identification of quantitative trait loci for kernel traits in a wheat cultivar Chuannong16
Source: BMC Genet. 2019 Oct 16;20:77. doi: 10.1186/s12863-019-0782-4 (PMC6796374; doi:10.1186/s12863-019-0782-4)

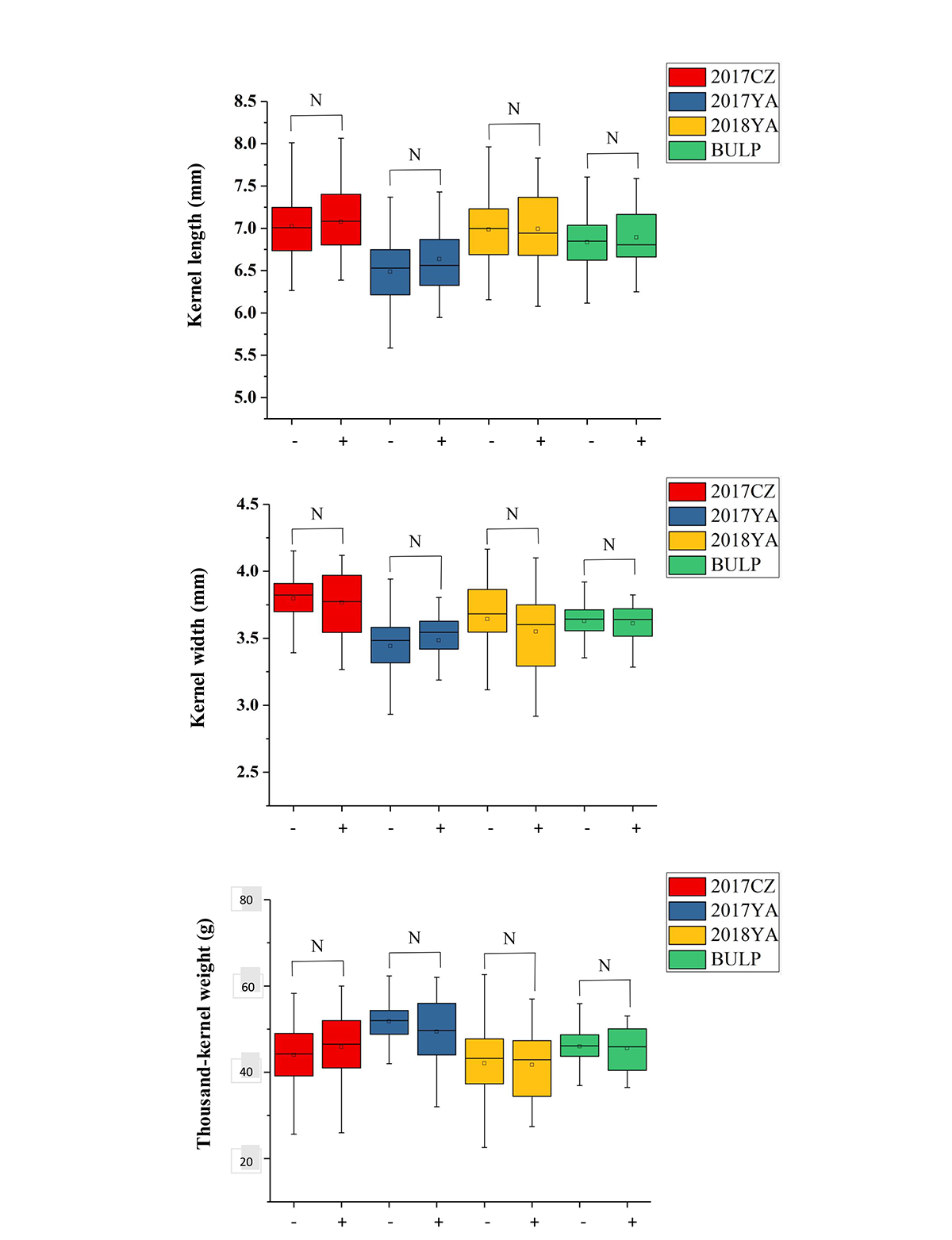

Supplement: Supplementary file 1 — Additional file 1: Figure S1. Effect of 1BL/1RS translocation on kernel traits. ‘-’ represents the homozygous lines carrying 1BS, ‘+’ represents the homozygous lines carrying 1RS, N represents no significant difference were detected. [file 12863_2019_782_MOESM1_ESM.tif]
